# Common variants in the *PARL* and *PINK1* genes increase the risk to leprosy in Han Chinese from South China

**DOI:** 10.1038/srep37086

**Published:** 2016-11-23

**Authors:** Dong Wang, Deng-Feng Zhang, Jia-Qi Feng, Guo-Dong Li, Xiao-An Li, Xiu-Feng Yu, Heng Long, Yu-Ye Li, Yong-Gang Yao

**Affiliations:** 1 Key Laboratory of Animal Models and Human Disease Mechanisms of the Chinese Academy of Sciences & Yunnan Province, Kunming Institute of Zoology, Kunming, Yunnan, 650223, China; 2 Department of Dermatology, the First Affiliated Hospital of Kunming Medical University, Kunming, Yunnan, 650032, China; 3 Kunming College of Life Science, University of Chinese Academy of Sciences, Kunming, Yunnan 650201, China; 4 Yuxi City Center for Disease Control and Prevention, Yuxi, Yunnan, 653100, China; 5 Wenshan Institute of Dermatology, Wenshan, Yunnan, 663000, China

## Abstract

Leprosy is a chronic infectious and neurological disease caused by *Mycobacterium leprae*, an unculturable pathogen with massive genomic decay and dependence on host metabolism. We hypothesized that mitochondrial genes *PARL* and *PINK1* would confer risk to leprosy. Thirteen tag SNPs of *PARL* and *PINK1* were analyzed in 3620 individuals with or without leprosy from China. We also sequenced the entire exons of *PARL, PINK1* and *PARK2* in 80 patients with a family history of leprosy by using the next generation sequencing technology (NGS). We found that *PARL* SNP rs12631031 conferred a risk to leprosy (*P*_*adjusted*_ = 0.019) and multibacillary leprosy (MB, *P*_*adjusted*_ = 0.020) at the allelic level. rs12631031 and rs7653061 in *PARL* were associated with leprosy and MB (dominant model, *P*_*adjusted*_ < 0.05) at the genotypic level. *PINK1* SNP rs4704 was associated with leprosy at the genotypic level (*P*_*adjusted*_ = 0.004). We confirmed that common variants in *PARL* and *PINK1* were associated with leprosy in patients underwent NGS. Furthermore, PARL and PINK1 could physically interact with each other and were involved in the highly connected network formed by reported leprosy susceptibility genes. Together, our results showed that *PARL* and *PINK1* genetic variants are associated with leprosy.

Leprosy is a chronic infectious disease which has affected mankind for more than 4,000 years^1^. Although the number of new cases of leprosy globally decreased to 213,899 patients in 2014^2^, the disease is still a significant threat to public health in many parts of the world. The pathogen, *Mycobacterium leprae (M. leprae*), is an obligate intracellular parasite and primarily affects the skin and peripheral nerves^3^. When compared against its close relative, *M. tuberculosis*, the genome of *M. leprae* shows an extremely eroded evolution, which has led to nearly half of the functional genes (especially in the metabolic pathways) undergoing inactivation or pseudogenation^4^,^5^,^6^. This marked reduction in the number of working genes might be the primary reason why *M. leprae* has a long half-life *in vivo* and cannot be cultured *in vitro*. As a result, the provision of energy metabolites and nutritional products by the host has become essential to the survival of *M. leprae*.

Mitochondria are crucial organelles involved in the cellular energy supply, regulation of apoptotic signals and autophagy, and defenses against pathogenic microbe invasion^7^,^8^,^9^. Recent studies showed that the host mitochondria might play important roles in *M. leprae* infection. A lower expression of several mitochondrial genes was observed in nerve biopsies from leprosy patients compared to non-leprous individuals using a microarray assay^10^. We also found a significantly increased mtDNA copy number in lepromatous leprosy patients^11^ compared with controls. The mitochondrial outer membrane protein, LRRK2, has been identified by a genome-wide association study (GWAS) as one of the leprosy susceptibility genes in Han Chinese population^12^, and this was confirmed by our recent case-control study^13^ and other studies^14^,^15^, although the associated *LRRK2* SNPs or their effects were different in these studies. Most recently, we provided solid evidence to show that the *OPA1* gene, encoding an mitochondrial inner membrane protein, was associated with leprosy susceptibility possibly by affecting mitochondrial function and antimicrobial pathways^16^. All these lines of evidence support our previous hypothesis that mitochondrial function may affect host susceptibility to *M. leprae* and the onset of clinical leprosy^11^.

The *presenilins-associated rhomboid-like (PARL)* gene is located on chromosome 3q27 and consists of ten exons. PARL is a mitochondrial membrane protein and is a key regulator of mitochondrial integrity and function, such as mitochondrial morphology, apoptosis and glucose metabolism^17^,^18^,^19^. PARL can interact with OPA1 during apoptosis by regulating apoptotic cristae remodeling and cytochrome c release^20^. Moreover, PARL together with OPA1 can control mitochondrial morphology^19^ and participate in mitochondrial adaptation to heat shock^21^. Genetic variants in the *PARL* gene can influence mitochondrial content^22^ and susceptibility to Parkinson’s disease^23^, type 2 diabetes^24^ and LHON^25^, although there were some negative reports^26^,^27^.

The PTEN induced putative kinase 1 (PINK1) is a serine/threonine kinase protein that is localized in mitochondria^28^. PINK1 knockout mice had mitochondrial dysfunction and increased sensitivity to oxidative stress^29^. Moreover, PINK1 could phosphorylate Parkin, leading to the activation of E3 ligase and the NF-κB signaling pathway^30^. The cleavage of PINK1 was mediated by PARL and this was affected by mitochondrial membrane potential^31^. This scenario negatively regulated the PINK1- and PARK2/Parkin-dependent mitophagy^32^. Mutations in *PINK1* have been reported to be associated with Parkinson’s disease^28^,^33^ and schizophrenia^34^, but there was a controversy^27^.

In this study, we aimed to investigate the possible association of genetic variants in the *PARL* and *PINK1* genes with leprosy in Han Chinese. Our results provided several lines of evidence showing that *PARL* and *PINK1* confer genetic susceptibility to leprosy.

## Results

### Association of *PARL* and *PINK1* SNPs with leprosy *per se* and multibacillary patients

**Figure 1:**
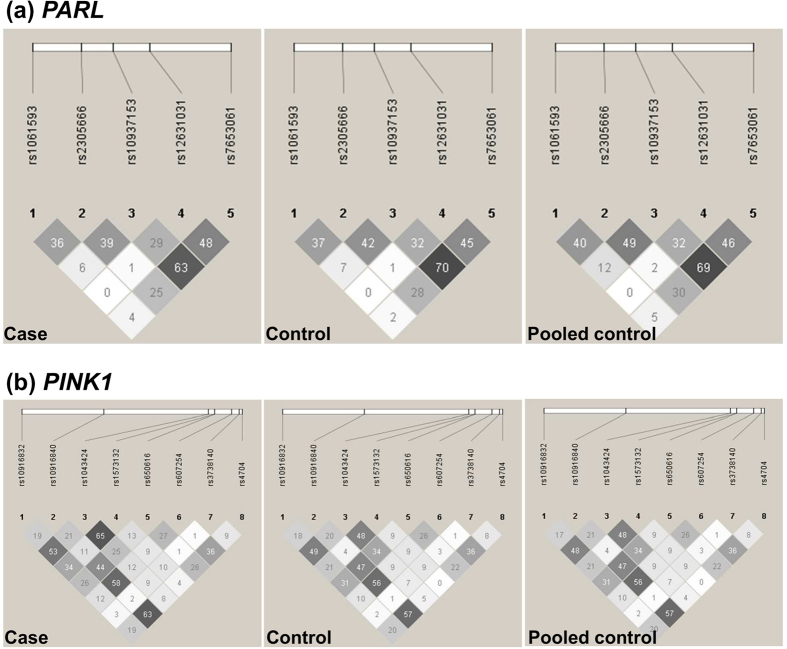
The linkage disequilibrium (LD) structures of *PARL* (**a**) and *PINK1* (**b**) in leprosy patients and healthy controls from the Yuxi Prefecture and pooled control samples. Black squares represented high LD as measured by *r*^*2*^, gradually coloring down to white squares of low LD. The individual square showed the *r*^*2*^ value for each SNP pair (*r*^*2*^ value is multiplied by 100). The pooled control samples contained the reported Han Chinese without leprosy from Hunan Province (N = 984), Shanghai (N = 1526)^27^, and the Yuxi control samples in this study (Yuxi).

The minor allele frequencies (MAF) of the SNPs analyzed in this study ranged from 5.8% to 48.4% (Table 1). The power to detect an odds ratio (OR) value as 1.6 for risk allele was expected to be above 77.0% (Fig. S1). SNPs rs10937153, rs1573132 and rs607254 were not in Hardy-Weinberg equilibrium in controls (Table S1, *P* < 0.05) and were excluded in the following analyses. The allele and genotype frequencies of the 10 SNPs in 527 leprosy patients, 583 healthy subjects from the Yuxi Prefecture, Yunnan Province, and pooled 3093 leprosy-unaffected controls were listed in Tables 1 and 2. We constructed the linkage disequilibrium (LD) map of all the tested SNPs in the Yuxi leprosy cases, Yuxi controls and pooled leprosy-unaffected controls (Fig. 1), and observed similar LD structures for these populations. We further performed the principal component (PC) analysis for the studied populations based on the observed genotype frequencies of the 10 SNPs, together with data of the CHB, CHD, JPT, CEU populations from the HapMap data set^35^. The Yuxi leprosy patient, Yuxi controls and the reported controls from Hunan Province and Shanghai were clustered together, suggesting no substantial population substructure between the cases and controls (Fig. S2).

The RegulomeDB database was used to annotate the analyzed SNPs^36^. Except for rs2305666 and rs1043424, the other SNPs showed a signal as DNase I hypersensitivity site. SNPs rs10916840 and rs4704 were located in transcription factor binding sites, and rs1061593 showed an eQTL effect (Table 1). Two *PARL* SNPs showed an association with leprosy *per se* (rs12631031-A allele, OR = 1.189, 95% CI [1.029–1.381], *P* = 0.019; rs12631031, *P*_dominant_ = 0.033; rs7653061, *P*_dominant_ = 0.027) and MB (rs12631031-A allele, OR = 1.251, 95% CI [1.036–1.510], *P* = 0.020; rs12631031, *P*_dominant_ = 0.019; rs7653061, *P*_dominant_ = 0.023) when compared with healthy control population from the same region or with the pooled control populations (Tables 1 and 2). One *PINK1* SNP (rs4704) showed an association with leprosy *per se* when compared with healthy control population from the Yuxi area (*P*_dominant_ = 0.033), and the association survived (TT vs. TC vs. CC, *P*_genotypic_ = 0.004; *P*_dominant_ = 0.027) when we compared the patients with the pooled controls (Table 2).

Haplotypes were reconstructed for four *PARL* SNPs (rs1061593-rs2305666-rs12631031-rs7653061) and four *PINK1* SNPs (rs10916832-rs10916840-rs1043424-rs4704; SNPs rs650616 and rs3738140 were excluded because these two SNPs were not genotyped in the pooled populations). There were no associations of *PARL* haplotypes or *PINK1* haplotypes with leprosy (cases versus pooled control samples, global *P-*value > 0.05). We observed no significant difference of haplotype distribution frequencies between the cases and controls (Table S2).

### Deep sequencing of *PARL* and *PINK1* exons identified an association of coding variants with leprosy

To identify whether there are any other rare (allele frequency < 1%) or common variants that would confer risk to leprosy, we performed targeted gene sequencing (including the flanking region of the gene) for *PARL, PINK1* and *PARK2* in 80 leprosy patients from the Wenshan Prefecture, Yunnan Province, and compared to the CHB data in 1000 Genomes dataset^37^. Although we did not find any rare variants of *PARL* and *PINK1* to be associated with leprosy (*P *> 0.05; partially due to the small sample size), one missense variant (rs3732581 [p.V212L], *P* = 6.434 × 10^−5^) and one synonymous variant (rs13091 [p.H216], *P* = 1.058 × 10^−4^) in *PARL* and one synonymous variant (rs45530340, [p.L63], *P* = 2.668 × 10^−4^) in *PINK1* were significantly associated with leprosy *per se* (Table S3). It should be mentioned that the comparison might be biased as we compared the Wenshan sample to the CHB sample (103 Han Chinese from Beijing) from the 1000 Genomes dataset^37^ and the samples were not geographically matched. Further *in silico* program affiliated predication analysis showed that no missense variants in *PARL, PINK1* and *PARK2* were predicted to be pathogenic (Table S3).

### The risk SNPs affected leprosy-related gene expression in human tissues

**Figure 2:**
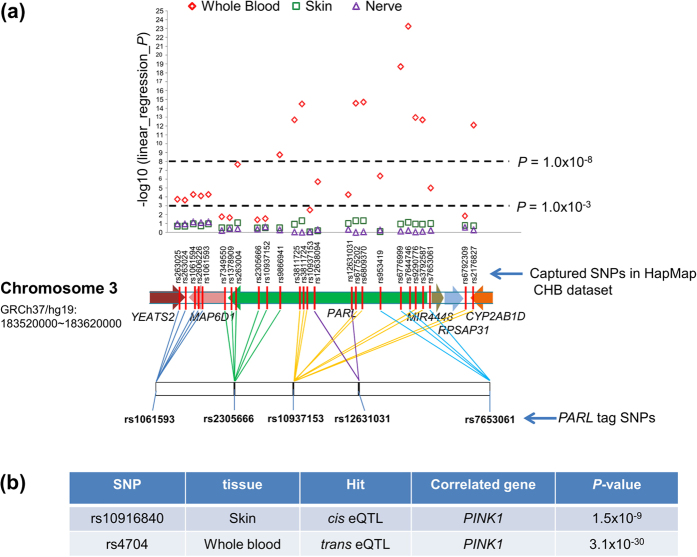
eQTL analysis of the *PARL* and *PINK1* genes. *cis* and *trans* eQTL of the *PARL* and *PINK1* tag SNPs in human blood, skin and nerve tissues were identified by using the GTEx (http://www.gtexportal.org/home)^38^ and HaploReg dataset (http://www.broadinstitute.org/mammals/haploreg/haploreg.php)^54^.

We tested the expression quantitative trait loci (eQTLs) of 34 SNPs (including 5 index tag SNPs and 21 captured *PARL* SNPs from the HapMap database^35^, and 8 tag SNPs in *PINK1*) in leprosy-related human blood, skin and nerve tissues from the Genotype-Tissue Expression project (GTEx, http:// www.gtexportal.org/ home/38). We found that 18 of 26 *PARL* SNPs were significant *cis* eQTLs in whole blood (*P *< 1.0 × 10^−4^). Among them, 10 of 26 SNPs were remarkably significant (*P *< 1.0 × 10^−8^). Notably, SNP rs7644746 that was tagged by the risk SNP rs7653061 reached a *P* value of 5.6 × 10^−24^ in blood (Fig. 2a). *PINK1* SNP rs10916840 was a *cis* eQTL in skin tissue (1.5 × 10^−9^) based on the GTEx dataset^38^, whereas rs4704 was a significant *trans* eQTL in whole blood (3.1 × 10^−30^; Fig. 2b). Both SNPs affected the *PINK1* mRNA expression level.

The specific expression pattern of *PARL* and *PINK1* were checked in a variety of human tissues from the BioGPS^39^ (http://biogps.org/#goto=welcome; Figures S3 and S4). We noticed that *PARL* mRNA expression level was extremely high in immune cells, but *PINK1* had an extremely high mRNA expression in central nervous system. We observed a significantly differential mRNA expression of *PINK1*, but not *PARL*, in leprosy skin lesions of 66 patients from the Gene Expression Omnibus dataset (GEO; http://www.ncbi.nlm.nih.gov/geo/query/acc.cgi?acc=GSE74481)^40^ (Table S4).

### Protein interaction network analysis showed an active interaction of PARL with leprosy risk genes

**Figure 3:**
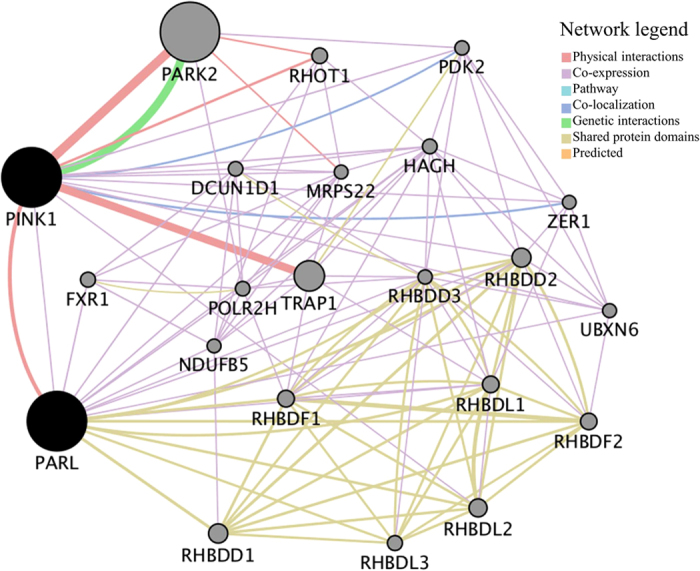
Protein interaction network of the *PARL* and *PINK1* genes. PARL can directly interact with PINK1 according to the GeneMANIA database (http://genemania.org/)^41^. The minimum required interaction score is >0.7 and the line thickness indicates the strength of data support.

To evaluate the protein interaction with PARL and PINK1, we used the GeneMANIA prediction server^41^ and identified that PARL could physically interacted and co-expressed with PINK1. PARL and PINK1 could directly or indirectly interact with many proteins, such as FXR1, NDUFB5, TRAP1 and PARK2 (Fig. 3). Note that PINK1 directly interacted with PARK2, which was identified as a leprosy risk gene in several populations^42^,^43^,^44^. However, our NGS analysis for the *PARK2* gene revealed no association of this gene with leprosy though we observed positive associations between *PARL* and leprosy or between *PINK1* and leprosy in this relatively small sample. This observation was consistent with a previous report for no association of *PARK2* SNPs with leprosy in Han Chinese population^45^. However, it should be noted that our exon sequencing of the *PARK2* gene did not cover its promoter region, and we could not exclude a possibility that there existed leprosy-associated SNP(s).

To discern whether PARL and PINK1 participated in molecular networks that contain proteins encoded by leprosy susceptibility genes, we constructed the protein interaction network of PARL, PINK1 and the reported 228 leprosy-associated genes (Table S5; ref. 46 and references therein) by the GeneMANIA^41^. We found that PARL and PINK1 could physically interacted, co-expressed and genetically interacted with those proteins of the reported leprosy susceptibility genes, such as *OPA1, PARK2, HLA-A, HLA-DRA, HLA-DQB*, and *IL10RA* (ref. 46 and references therein) (Figure S5).

## Discussion

Leprosy is a complex infectious and neurological disease, with impairment of both the immune and peripheral nerve systems during the infection^3^. Host genetic background would affect the susceptibility to leprosy, because of the genomic decay of *M. leprae*, and its completely parasitic mode. Genetic studies, especially recent GWAS in Han Chinese^12^, have suggested an important role for host genetic effect on leprosy susceptibility, although the exact mechanism was still unknown^47^,^48^. Mitochondria play important roles in cellular energy supply, cell signaling, mitophagy and anti-microbe immune responses^7^,^8^,^9^. It is therefore reasonable to believe that genes involved in mitochondrial function would affect the host response to microbe infection. Indeed, we recently provided evidence that genetic variants of the mitochondrial genes, like *LRRK2* and *OPA1* were associated with leprosy^13^,^16^, although three mitochondrial-related antimicrobial/antiviral immune genes (*MAVS, MITA* and *MFN2*) showed no evidence to be associated with leprosy^49^. In this study, we found that two mitochondrial genes, *PARL* and *PINK1*, conferred genetic susceptibility to leprosy *per se* and/or multibacillary leprosy.

Among the analyzed tag SNPs in the *PARL* and *PINK1* genes, three SNPs (rs12631031 and rs7653061 of *PARL*; rs4704 of *PINK1*) were associated with leprosy *per se* and/or MB. Furthermore, deep sequencing of the *PARL, PINK1* and *PARK2* genes in a relatively small sample identified two *PARL* variants (rs3732581 [p.V212L], rs13091 [p.H216]) and one *PINK1* variant (rs45530340, [p.L63]) that were associated with leprosy. We found no variant in the coding region of the *PARK2* gene to be linked with leprosy. There is a possibility that promoter variant in this gene might confer risk to leprosy and this awaits future study, as the promoter region was not covered by the current exon sequencing. It was well known that genetic variation could influence gene expression^50^,^51^, therefore we performed eQTL analysis to elucidate whether these leprosy risk variants altered the *PARL* and *PINK1* mRNA expression. We observed that two risk SNPs of *PARL* and all their captured SNPs were *cis* eQTLs for *PARL* mRNA expression in human blood (*P* value from 5.6 × 10^−5^ to 5.6 × 10^−24^), and two risk SNPs of *PINK1* were *cis* and *trans* eQTLs for *PINK1* mRNA expression in skin (*P* = 1.5 × 10^−9^) and blood (*P* = 3.1 × 10^−30^), respectively. Nevertheless, we only found a significantly different expression of *PINK1* mRNA, but not *PARL* mRNA, between leprotic lesions (leprosy *per se* or its subtypes) and control tissues based on the re-analysis of reported datasets^10^,^40^. The exact reason for the discrepancy of *PARL* mRNA expression remains unknown and awaits future study.

In our recent studies, we identified an association of two mitochondrial genes (*LRRK2* and *OPA1*) with leprosy^13^,^16^. Although there was no positive interaction among *PARL, PINK1, OPA1* and *LRRK2* SNPs (Table S6) based on our recently reported data^13^,^16^ and current data, the positive associations of these four genes with leprosy suggested that mitochondrial related genes should play active roles in leprosy. The protein interaction network analysis supported this speculation, as we found that the other mitochondrial genes (*MCCD1, SDHD, SNCA*, and *VARS2*) could interact with the reported leprosy susceptibility genes (Table S5). Whether these genes play their roles by directly affecting mitochondrial function, or by participating in other signaling pathways, and then affect leprosy susceptibility, is still an open question.

This study had two limitations. First, the Wenshan population analyzed by the NGS was relatively small, and we compared this population to the CHB data in 1000 Genomes dataset^37^, which might lead to a biased result as the samples were not well matched. For the Yuxi sample, the coverage of common *PARL* and *PINK1* SNPs might not be sufficient. Second, we did not perform functional assays to characterize the role of *PARL, PINK1* and their interactions with previously reported mitochondrial risk genes such as *OPA1*^16^ and *LRRK2*^13^ during *M. leprae* infection.

In summary, we found that common variants of the mitochondrial genes *PARL* and *PINK1* would confer risk to leprosy *per se* and/or MB. Combining the reported results^13^,^16^,^46^ and the protein interaction network analysis, we found that *PARL* and *PINK1* were participated in a highly connected network formed by the reported leprosy risk genes (ref. 46 and references therein). Future studies are needed to validate the association in independent populations and to explore the underlying mechanism during leprosy onset and progression.

## Materials and Methods

### Study subjects

This study was carried out in 1,110 individuals from the Yuxi Prefecture, Yunnan Province: 527 individuals were leprosy patients (onset age from 2 to 67 years, mean age: 24.7 ± 12.3 years; male/female ratio = 387/140; multibacillary/paucibacillary = 279/ 248); 583 individuals were healthy control subjects from the same geographic area (age from 4 to 88 years, mean age: 36.0 ± 15.5 years; male/female ratio = 365/ 218). These samples had been analyzed for potential associations of other genes with leprosy in our previous studies^13^,^52^. A total of 80 unrelated leprosy patients (38 lepromatous leprosy [LL] patients and 42 tuberculoid leprosy [TT] patients) with a family history of disease (each family has at least two leprosy patients) were collected from the Wenshan Prefecture, Yunnan Province. In brief, the diagnosis of leprosy patients was based on clinical and histopathological features, as well as the bacteriological index if available. A total of 2,510 unaffected Han Chinese from South China (including 504 schizophrenia cases and 480 healthy controls from Hunan Province and 624 schizophrenia cases and 902 healthy controls from Shanghai) that were analyzed for 5 *PARL* SNPs and 4 *PINK1* SNPs in our recent study^27^ were included in this study for comparison, as we found no association between *PARL* and *PINK1* variants and schizophrenia in these sample groups^27^. All healthy individuals and the reported schizophrenia patients had no history of leprosy, HIV infection, and tuberculosis. Written informed consents conforming to the tenets of the Declaration of Helsinki were obtained from each participant or the appointed guardians of the patients (for those who were unable to provide informed consent at the time of blood collection) prior to the study. The experimental methods were carried out in accordance with the approved guidelines. The institutional review board of the Kunming Institute of Zoology (KIZ) approved all experimental protocols of this study.

### SNP selection, genotyping and NGS

Genomic DNA was extracted from whole blood by using the AxyPrep™ Blood Genomic DNA Miniprep Kit (Axygen, USA). We selected five *PARL* tag SNPs (rs1061593, rs2305666, rs10937153, rs12631031, rs7653061; Figure S6) and eight *PINK1* tag SNPs (rs10916832, rs10916840, rs1043424, rs1573132, rs650616, rs607254, rs3738140, rs4704; Figure S7) that were located in a region spanning *PINK1* to *DDOST* based on the linkage disequilibrium (LD) pattern of the analyzed genes using the international HapMap project data set (www.hapmap.ncbi.nlm.nih.gov/, Phase 3, CHB^35^). The potential roles of SNPs, e.g. affecting transcription factor binding sites or enacting other regulatory factor / mechanism, were estimated by referring to the RegulomeDB dataset (http://www.regulomedb.org/)^36^. All SNPs were genotyped in the cases and controls from the Yuxi Prefecture by using the SNaPshot assay (Table S7) as described in our previous studies^27^,^52^ at the Kunming Biological Diversity Regional Center of Instruments, KIZ. For the NGS of the *PARL, PINK1* and *PARK2* genes in 80 leprosy patients from the Wenshan Prefecture, we used the same approach as described in our recent study^53^.

### PC analysis, expression and expression quantitative trait loci (eQTL) analysis

PC analysis was performed using the genotype frequencies of 10 tag SNPs (three SNPs were excluded due to the deviation of the Hardy-Weinberg equilibrium [HWE]) to show the overall clustering pattern of the 8 populations (leprosy and control populations from the Yuxi Prefecture of Yunnan Province, unaffected Han Chinese populations from Hunan Province and Shanghai^27^, CHB [136 Han Chinese in Beijing], CHD [109 Chinese in Metropolitan Denver, Colorado], JPT [113 Japanese in Tokyo, Japan], and CEU [113 Utah residents with Northern and Western European ancestry] populations from the HapMap database^35^) by using the POPSTR software (http://harpending.humanevo.utah.edu/popstr/).

We performed eQTL analysis in different human tissues by using two publically available expression data sets^10^,^40^. We first investigated whether the *PARL* and *PINK1* variants affect gene expression in human whole blood, brain and skin tissues using the Genotype-Tissue Expression project (GTEx, http://www.gtexportal.org/home/38) data and the HaploReg dataset (http://www.broadinstitute.org/mammals/haploreg/haploreg.php)^54^. We also considered the overall expression profiling of these two genes in the BioGPS database (http://biogps.org/#goto=welcome)^39^.

We reanalyzed the largest microarray data regarding leprosy skin lesions, including 24 MB (10 mid-borderline leprosy [BB] + 10 borderline lepromatous [BL] + 4 lepromatous [LL]), 20 PB (10 tuberculoid [TT] + 10 borderline-tuberculoid [BT]), 14 type I reaction (R1), and 10 type II reaction (R2) patients, and normal skin biopsies from 9 healthy individuals. The data was retrieved from GEO under accession series GSE74481 (http://www.ncbi.nlm.nih.gov/geo/query/acc.cgi?acc=GSE74481)^40^.

### Protein interaction network analysis

To construct the potential protein interaction network of PARL and PINK1, and to test whether the *PARL* and *PINK1* genes interact with the other leprosy risk genes as compiled in our recent study (ref. 46 and references therein), we performed interaction network analysis by referring to a high-confidence protein interaction database GeneMANIA (http://www.genemania.org/)^41^.

### Statistical analysis

The genotyping call rate of each tag SNP was above 98.7% in our subjects. Cases and controls were compared on the basis of the frequencies of genotypes and alleles. We randomly selected about 2% of samples for direct sequencing and confirmed 100% of consistence with the SNaPshot genotyping results. Power calculations were performed using Quanto software^55^. Linkage disequilibrium (LD) structure was determined using Haploview 4.2 56. Deviation from the HWE, haplotype comparison and SNP-SNP interaction were performed by using PLINK v1.07 57. The significant SNPs were further calculated by using the logistic regression, with an adjustment for sex. We predicted the potential pathogenicity of variants in the *PARL, PINK1* and *PARK2* genes identified by the NGS by using an *in silico* program affiliated prediction, following the procedure described in our recent study (ref. 53 and references therein). A *P* value < 0.05 was considered to be statistically significant.

## Additional Information

**How to cite this article**: Wang, D. *et al*. Common variants in the *PARL* and *PINK1* genes increase the risk to leprosy in Han Chinese from South China. *Sci. Rep.* **6**, 37086; doi: 10.1038/srep37086 (2016).

**Publisher's note:** Springer Nature remains neutral with regard to jurisdictional claims in published maps and institutional affiliations.

## Supplementary Material

supplementary Data

## Figures and Tables

**Table 1:** Allele frequencies of 4 *PARL* SNPs and 6 *PINK1* SNPs in 527 leprosy patients and 583 healthy controls from the Yuxi Prefecture of Yunnan Province, and in 3093 pooled Han Chinese across China.

SNP ID	Population*	Allele	MAF (control)	Leprosy *per se*			MB			PB			RegulomeDB Score^a^
				MAF	*P*^#^	OR (95%CI)	MAF	*P*^#^	OR	MAF	*P*^*#*^	OR (95%CI)	
rs1061593	Yuxi	A/G	0.478	0.484	0.777	1.024 (0.867-1.211)	0.477	0.966	0.996 (0.813-1.219)	0.492	0.601	1.058 (0.856-1.307)	1 f
	Pooled		0.479		0.801	1.017 (0.892-1.160)		0.896	0.988 (0.831-1.176)		0.601	1.050 (0.874-1.263)	
rs2305666	Yuxi	C/A	0.388	0.384	0.840	0.982 (0.827-1.167)	0.376	0.619	0.948 (0.770-1.169)	0.393	0.839	1.023 (0.822-1.272)	6
	Pooled		0.402		0.277	0.928 (0.810-1.062)		0.228	0.896 (0.749-1.072)		0.721	0.966 (0.798-1.169)	
rs12631031	Yuxi	A/G	0.299	0.303	0.814	1.022 (0.852-1.226)	0.314	0.522	1.074 (0.863-1.336)	0.291	0.761	0.965 (0.765-1.217)	5
	Pooled		0.267		**0.019**	1.189 (1.029-1.381)		**0.020**	1.251 (1.036-1.510)		0.252	1.126 (0.919-1.380)	
rs7653061	Yuxi	G/T	0.484	0.467	0.444	0.937 (0.793-1.107)	0.473	0.678	0.958 (0.783-1.173)	0.461	0.400	0.913 (0.739-1.129)	5
	Pooled		0.442		0.121	1.110 (0.973-1.266)		0.153	1.135 (0.954-1.351)		0.405	1.082 (0.899-1.302)	
rs10916832	Yuxi	C/T	0.342	0.349	0.750	1.029 (0.862-1.230)	0.362	0.422	1.091 (0.882-1.350)	0.333	0.734	0.962 (0.767-1.205)	5
	Pooled		0.34		0.560	1.042 (0.908-1.196)		0.281	1.104 (0.922-1.323)		0.785	0.973 (0.800-1.184)	
rs10916840	Yuxi	A/G	0.270	0.281	0.558	1.059 (0.874-1.284)	0.281	0.623	1.060 (0.840-1.337)	0.281	0.645	1.058 (0.831-1.348)	4
	Pooled		0.265		0.301	1.082 (0.932-1.256)		0.431	1.083 (0.888-1.319)		0.465	1.081 (0.877-1.333)	
rs1043424	Yuxi	C/A	0.363	0.357	0.783	0.976 (0.818-1.164)	0.362	0.961	0.995 (0.805-1.230)	0.352	0.677	0.954 (0.763-1.192)	7
	Pooled		0.375		0.279	0.927 (0.809-1.063)		0.543	0.946 (0.789-1.133)		0.319	0.907 (0.747-1.100)	
rs650616	Yuxi	A/G	0.437	0.468	0.149	1.133 (0.956-1.344)	0.457	0.427	1.087 (0.885-1.333)	0.480	0.112	1.189 (0.960-1.473)	5
rs3738140	Yuxi	A/G	0.067	0.058	0.380	0.856 (0.605-1.211)	0.052	0.225	0.763 (0.491-1.183)	0.065	0.864	0.963 (0.629-1.476)	5
rs4704	Yuxi	T/C	0.372	0.388	0.455	1.069 (0.898-1.273)	0.393	0.418	1.090 (0.885-1.344)	0.382	0.700	1.044 (0.838-1.302)	4
	Pooled		0.375		0.449	1.054 (0.921-1.206)		0.426	1.075 (0.900-1.284)		0.766	1.029 (0.851-1.245)	

MB – multibacillary leprosy; PB – paucibacillary leprosy; *P* - *P* value; OR – Odds Ratio; 95% CI – 95% confidence interval; MAF – minor allele frequency.

^*^Pooled - Pooled Han Chinese without leprosy, which contained the reported samples from Hunan Province (N = 984), Shanghai (N = 1526)^27^, and the Yuxi control samples in this study (Yuxi).

^#^*P* values < 0.05 were marked in bold and were recalculated by using the unconditional logistic regression, with an adjustment for sex.

^a^The RegulomeDB score was taken from http://www.regulomedb.org/:^36^ 1 f, eQTL + TF binding/DNase peak; 4, TF binding + DNase peak; 5, TF binding or DNase peak; 6, other; 7, No data.

**Table 2:** Comparison of the genotype frequencies of 4 *PARL* SNPs and 6 *PINK1* SNPs in 527 leprosy patients and 583 healthy controls from the Yuxi Prefecture of Yunnan Province, and in 3093 pooled unaffected Han Chinese.

SNP ID	Test model	No. of controls^*^	Leprosy *per se* vs. Controls		MB vs. Controls		PB vs. Controls	
			No.	*P*^*#*^	No.	*P*^*#*^	No.	*P*^*#*^
rs1061593	GENO (Yuxi | pooled)	132/293/158 | 703/1542/829	123/259/140	0.936 | 0.940	61/143/74	0.947 | 0.909	62/116/66	0.664 | 0.624
AA/GA/GG	DOM (Yuxi | pooled)	425/158 | 2948/3200	382/140	0.916 | 0.943	204/74	0.881 | 0.900	178/66	0.988 | 0.978
	REC (Yuxi | pooled)	132/451 | 2245/829	123/399	0.717 | 0.727	61/217	0.818 | 0.724	62/182	0.392 | 0.365
rs2305666	GENO (Yuxi | pooled)	86/278/216 | 494/1478/1098	70/256/190	0.788 | 0.345	35/138/104	0.677 | 0.318	35/118/86	0.928 | 0.834
CC/CA/AA	DOM (Yuxi | pooled)	364/216 | 1972/1098	326/190	0.886 | 0.644	173/104	0.932 | 0.554	153/86	0.734 | 0.946
	REC (Yuxi | pooled)	86/494 | 494/2576	70/446	0.551 | 0.145	35/242	0.389 | 0.131	35/204	0.946 | 0.557
rs12631031	GENO (Yuxi | pooled)	52/244/287 | 211/1220/1643	46/225/252	0.925 | 0.052	24/127/128	0.591 | 0.056	22/98/124	0.901 | 0.407
AA/AG/GG	DOM (Yuxi | pooled)	296/287 | 1431/1643	271/252	0.729 | **0.033**	151/128	0.357 | **0.019**	120/124	0.676 | 0.428
	REC (Yuxi | pooled)	52/531 | 211/2863	46/477	0.942 | 0.113	24/255	0.878 | 0.276	22/222	0.964 | 0.205
rs7653061	GENO (Yuxi | pooled)	136/292/155 | 601/1513/960	103/282/137	0.289 | 0.058	53/157/68	0.186 | 0.254	50/125/69	0.657 | 0.631
GG/GT/TT	DOM (Yuxi | pooled)	428/155 | 2114/960	385/137	0.898 | **0.027**	210/68	0.506 | **0.023**	175/69	0.618 | 0.337
	REC (Yuxi | pooled)	136/447 | 601/2473	103/419	0.147 | 0.923	53/225	0.158 | 0.845	50/194	0.373 | 0.722
rs10916832	GENO (Yuxi | pooled)	66/245/240 | 349/1367/1326	62/240/220	0.877 | 0.825	37/128/114	0.725 | 0.551	25/112/106	0.771 | 0.842
CC/CT/TT	DOM (Yuxi | pooled)	311/240 | 1716/1326	302/220	0.641 | 0.534	165/114	0.458 | 0.379	137/106	0.987 | 0.992
	REC (Yuxi | pooled)	66/485 | 349/2693	62/460	0.959 | 0.789	37/242	0.596 | 0.372	25/218	0.491 | 0.576
rs10916840	GENO (Yuxi | pooled)	39/212/287 | 212/1182/1632	32/217/251	0.413 | 0.184	19/111/135	0.791 | 0.635	13/106/116	0.286 | 0.169
AA/AG/GG	DOM (Yuxi | pooled)	251/287 | 1394/1632	249/251	0.311 | 0.121	130/135	0.522 | 0.349	119/116	0.308 | 0.176
	REC (Yuxi | pooled)	39/499 | 212/2814	32/468	0.588 | 0.621	19/246	0.968 | 0.920	13/222	0.381 | 0.390
rs1043424	GENO (Yuxi | pooled)	73/253/224 | 422/1434/1185	66/240/215	0.954 | 0.553	39/123/116	0.883 | 0.619	27/117/99	0.673 | 0.475
CC/CA/AA	DOM (Yuxi | pooled)	326/224 | 1856/1185	306/215	0.858 | 0.321	162/116	0.783 | 0.367	144/99	0.997 | 0.586
	REC (Yuxi | pooled)	73/477 | 422/2619	66/455	0.769 | 0.458	39/239	0.764 | 0.944	27/216	0.398 | 0.227
rs650616	GENO (Yuxi)	108/265/178	116/257/150	0.355	56/143/80	0.554	60/114/70	0.251
AA/AG/GG	DOM (Yuxi)	373/178	373/150	0.197	199/80	0.286	174/70	0.310
	REC (Yuxi)	108/443	116/407	0.298	56/223	0.872	60/184	0.112
rs3738140	GENO (Yuxi)	5/67/502	1/59/466	NA	1/27/251	NA	0/32/215	NA
AA/AG/GG	DOM (Yuxi)	72/502	60/466	NA	28/251	NA	32/215	NA
	REC (Yuxi)	5/569	1/525	NA	1/278	NA	0/247	NA
rs4704	GENO (Yuxi | pooled)	77/256/218 | 434/1416/1192	61/282/178	**0.033 | 0.004**	34/151/94	0.113 | 0.053	27/131/84	0.130 | 0.066
TT/TC/CC	DOM (Yuxi | pooled)	333/218 | 1850/1192	343/178	0.067 | **0.027**	185/94	0.099 | 0.071	158/84	0.195 | 0.169
	REC (Yuxi | pooled)	77/474 | 434/2608	61/460	0.268 | 0.119	34/245	0.475 | 0.339	27/215	0.279 | 0.180

MB – multibacillary leprosy; PB – paucibacillary leprosy; GENO: genotypic; DOM: dominant model; REC: recessive model; *P* - *P* value; OR – Odds Ratio; 95% CI – 95% confidence interval; *NA*– not available.

^*^Pooled - Pooled Han Chinese without leprosy, which contained the reported samples from Hunan Province (N = 984), Shanghai (N = 1526)^27^, and the Yuxi control samples in this study (Yuxi).

^#^*P* values < 0.05 were marked in bold and recalculated by using the unconditional logistic regression, with an adjustment for sex.
