# Supplementary material for: Common variants in the PARL and PINK1 genes increase the risk to leprosy in Han Chinese from South China
Source: Sci Rep. 2016 Nov 23;6:37086. doi: 10.1038/srep37086 (PMC5120299; doi:10.1038/srep37086)
Supplement: supplementary Data [file srep37086-s1.pdf]

Online supplementary data

Common variants in the *PARL* and *PINK1* genes increase the risk to leprosy in Han Chinese from South China

Dong Wang[1], Deng-Feng Zhang[1], Jia-Qi Feng[1, 2], Guo-Dong Li[1, 5], Xiao-An Li[3], Xiu-Feng Yu[4], Heng Long[4], Yu-Ye Li[2], Yong-Gang Yao[1, 5]*

[1] Key Laboratory of Animal Models and Human Disease Mechanisms of the Chinese Academy of Sciences & Yunnan Province, Kunming Institute of Zoology, Kunming, Yunnan, 650223, China
[2] Department of Dermatology, the First Affiliated Hospital of Kunming Medical College, Kunming, Yunnan, 650032, China
[3] Yuxi City Center for Disease Control and Prevention, Yuxi, Yunnan, 653100, China
[4] Wenshan Institute of Dermatology, Wenshan, Yunnan, 663000, China
[5] Kunming College of Life Science, University of Chinese Academy of Sciences, Kunming, Yunnan 650201, China

Corresponding author:
Dr. Yong-Gang Yao, Kunming Institute of Zoology, Chinese Academy of Sciences, Kunming, Yunnan 650223, China. Tel/Fax: 86-871-65180085; E-mail: yaoyg@mail.kiz.ac.cn

Table S1. Hardy-Weinberg equilibrium test for 5 *PARL* SNPs and 8 *PINK1* SNPs

**SNPs**	***P-value*** **(Yuxi control)**	***P-value*** **(Pooled controls)**
rs1061593	0.934	0.692
rs2305666	0.861	0.881
rs10937153	**0.016**	0.084
rs12631031	1.000	0.550
rs7653061	1.000	0.942
rs10916832	0.777	1.000
rs10916840	1.000	0.889
rs1043424	0.927	0.728
rs1573132	**0.000**	-
rs650616	0.604	-
rs607254	**0.008**	-
rs3738140	0.167	-
rs4704	0.927	0.617

Note: *P*-values less than 0.05 were marked in bold. The Yuxi control sample has 583 individuals. The pooled controls contained the reported data from Hunan Province (N = 984), Shanghai (N = 1526) [1], and the Yuxi control individuals in this study (Yuxi).

Table S2. Association of the *PARL* and *PINK1* haplotypes with leprosy in Han Chinese (527 cases versus 3093 unaffected control individuals)

**Haplotype** [a]	**Pooled Control**	**Case vs. Control**		**MB vs. Control**		**PB vs. Control**	
	**Frequency**	**Frequency**	***P***	**Frequency**	***P***	**Frequency**	***P***
PARL							
G-A-G-T	0.352	0.334	0.271	0.320	0.143	0.350	0.900
A-C-G-G	0.136	0.125	0.348	0.125	0.475	0.125	0.500
A-C-A-G	0.134	0.150	0.175	0.140	0.658	0.160	0.109
A-A-G-T	0.125	0.136	0.348	0.144	0.220	0.127	0.898
G-A-A-G	0.118	0.139	0.061	0.157	0.009	0.119	0.947
A-C-G-T	0.088	0.074	0.160	0.072	0.205	0.078	0.447
G-C-G-G	0.032	0.030	0.728	0.028	0.637	0.032	0.998
G-C-A-G	0.015	0.012	0.452	0.014	0.794	0.010	0.374
**Global *P* value** [b]			0.220		0.136		0.786
PINK1							
T-G-C-T	0.087	0.067	0.033	0.066	0.092	0.068	0.153
T-A-A-T	0.265	0.285	0.182	0.286	0.307	0.285	0.344
C-G-A-T	0.021	0.029	0.111	0.032	0.086	0.025	0.543
C-G-C-C	0.288	0.290	0.924	0.298	0.634	0.280	0.716
C-G-A-C	0.031	0.030	0.781	0.031	0.963	0.028	0.694
T-G-A-C	0.308	0.300	0.617	0.288	0.340	0.313	0.784
**Global *P* value** [b]			0.145		0.218		0.672

[a] The order of *PARL* SNPs in each haplotype is rs1061593 - rs2305666 - rs12631031 - rs7653061 and *PINK1* SNPs in each haplotype is rs10916832 - rs10916840 - rs1043424 - rs4704. Three SNPs in *PARL* (rs10937153) and *PINK1* (rs1573132 and rs607254) were excluded from the analysis due to deviation from the Hardy-Weinberg equilibrium in the Yuxi control individuals ($P < 0.05$). SNPs rs650616 and rs3738140 were excluded because of no genotype information in the reported samples [1].

[b] Global *P* value was calculated by using the Chi-square test.

Table S3. The list of variants in the exon and flanking regions of the *PARL*, *PINK1* and *PARK2* genes in 80 leprosy patients underwent NGS

Chr.	Position	SNP ID [a]	Gene	Function	Ref.	Alt.	Residue change	Damaging predication [b]	Allele counts in 80 leprosy patients	Allele counts in CHB	*P*-value [c]	OR
chr3	183547346	rs371236566	*PARL*	utr-3	C	T	-	-	1/160	NA	NA	NA
chr3	183558402	rs3732581	*PARL*	missense	C	G	p.V212L	Tolerated	44/160	99/206	$\mathbf{6.434\times10^{-5}}$	0.410
chr3	183560195	rs13091	*PARL*	synonymous	A	G	p.H216	-	45/160	99/206	$\mathbf{1.058\times10^{-4}}$	0.423
chr1	20960230	rs45530340	*PINK1*	synonymous	C	T	p.L63	-	30/150	14/206	$\mathbf{2.668\times10^{-4}}$	3.429
chr1	20960442	rs117438827	*PINK1*	intron	G	A	-	-	3/150	7/206	0.528	0.580
chr1	20960453	rs377219724	*PINK1*	intron	G	C	-	-	3/152	1/206	0.316	4.128
chr1	20964328	rs2298298	*PINK1*	intron	A	G	-	-	137/160	171/206	0.565	1.219
chr1	20964332	rs371971165	*PINK1*	spliceSite	C	T	-	-	1/160	NA	NA	NA
chr1	20970949	rs199769220	*PINK1*	intron	T	C	-	-	1/160	1/206	1.000	1.289
chr1	20972048	rs3131713	*PINK1*	intron	G	A	-	-	131/160	171/206	0.783	0.925
chr1	20972111	rs3738136	*PINK1*	missense	G	A	p.A340T	Tolerated	38/160	54/206	0.628	0.877
chr1	20975154	rs370380947	*PINK1*	intron	G	A	-	-	1/160	NA	NA	NA
chr1	20975463	rs2298300	*PINK1*	intron	T	C	-	-	6/160	9/206	0.798	0.853
chr1	20976950	rs527565484	*PINK1*	synonymous	A	C	p.A504	-	1/160	NA	NA	NA
chr1	20977000	rs1043424	*PINK1*	missense	A	C	p.N521T	Tolerated	48/160	85/206	0.029	0.610
chr1	20977154	.	*PINK1*	synonymous	C	G	p.L572	-	1/160	NA	NA	NA
chr1	20977221	rs686658	*PINK1*	utr-3	A	T	-	-	134/160	171/206	0.888	1.055
chr1	20977224	rs115768147	*PINK1*	utr-3	G	A	-	-	6/160	9/206	0.798	0.853
chr6	161807855	rs1801582	*PARK2*	missense	C	G	p.V231L	Tolerated	29/160	15/206	0.002	2.819
chr6	161815583	rs3924680	*PARK2*	intron	C	T	-	-	4/160	NA	NA	NA
chr6	161966429	rs566245804	*PARK2*	intron	C	G	-	-	1/160	NA	NA	NA

chr6	162137099	rs112078090	*PARK2*	intron	CATCTT	C	-	-	32/160	38/206	0.789	1.105
chr6	162137147	rs77332950	*PARK2*	intron	C	T	-	-	3/160	11/206	0.104	0.339
chr6	162199393	rs12207168	*PARK2*	intron	A	G	-	-	69/160	76/206	0.238	1.297
chr6	162622197	rs1801474	*PARK2*	missense	C	T	p.S167N	Tolerated	50/160	75/206	0.319	0.794
chr6	162622304	rs4709583	*PARK2*	intron	A	G	-	-	159/160	201/206	0.237	3.955
chr6	162863033	rs9347641	*PARK2*	intron	C	A	-	-	35/160	23/206	0.006	2.228
chr6	162864317	rs2075923	*PARK2*	intron	A	G	-	-	29/160	31/206	0.478	1.250
chr6	162864528	rs201760977	*PARK2*	intron	A	G	-	-	1/160	1/206	1.000	1.289

Chr, Chromosome; Ref, Reference allele; Alt, Alternate allele; CHB, 103 Han Chinese from Beijing in the 1000 Genomes dataset [2]; OR, Odds ratio; NA, no data available.

[a] Among the 13 SNPs that were genotyped in this study, only one missense variant (rs1043424) was captured by the NGS.

[b] Missense variants are rated as damaging when at least two of five prediction algorithms (SIFT [3,4], PolyPhen2 HumDiv, PolyPhen2 HumVar [5], LRT [6] and MutationTaster [7]) suggesting a potential deleterious effect, otherwise the variants are rated as tolerated.

[c] *P*-values were calculated by using the Fisher's exact test.

Table S4. mRNA expression levels of the *PARL* and *PINK1* genes in leprosy skin lesions

Gene	Control vs. MB			Control vs. PB			Control vs. R1			Control vs. R2		
	adj. *P*. Val	*P*-Value	logFC	adj.*P*.Val	*P*-Value	logFC	adj.*P*.Val	*P*-Value	logFC	adj.*P*.Val	*P*-Value	logFC
PARL	2.38E-03	8.19E-04	0.300	8.02E-02	4.65E-02	0.141	5.07E-03	2.52E-03	0.234	1.53E-02	7.43E-03	0.254
PINK1	1.51E-04	3.23E-05	-0.496	6.49E-05	1.13E-05	-0.671	2.49E-06	5.69E-07	-0.675	3.97E-05	7.44E-06	-1.043

Note: The microarray expression data were retrieved from GEO according to accession series GSE74481 (http://www.ncbi.nlm.nih.gov/geo/query/acc.cgi?acc=GSE74481) [8]. This dataset contains skin biopsies of 24 MB (10 mid-borderline leprosy [BB] + 10 borderline lepromatous [BL] + 4 lepromatous [LL]), 20 PB (10 tuberculoid [TT] + 10 borderline-tuberculoid [BT]), 14 type I reaction (R1), 10 type II reaction (R2) patients, and 9 healthy individuals.

adj. *P*. Val – adjusted *P*-value; logFC –log2 Fold Change

Table S5. Reported leprosy susceptible genes used for constructing the PPI network

Gene	Gene	Gene	Gene	Gene	Gene
ADAMTSL1	*CCL5*	*HLA-DMA*	*IL4R*	*MASP2*	*RAB32*
AGER	*CD209*	*HLA-DMB*	*IL5RA*	*MBL2*	*RASSF8*
AGPAT1	*CDC42BPG*	*HLA-DOA*	*IL6*	*MCCD1*	*RDBP*
AIF1	*CDSN*	*HLA-DOB*	*IL6R*	*MEN1*	*RGNEF*
ALS2CL	*CFB*	*HLA-DPA1*	*IRGM*	*MICA*	*RIPK2*
APOE	*CFH*	*HLA-DPB1*	*KIR2DL1*	*MICB*	*RNF5*
APOH	*CLIC1*	*HLA-DQA1*	*KIR2DL2*	*MIR125A*	*SDHD*
APOM	*COL3A1*	*HLA-DQA2*	*KIR2DL3*	*MIR146A*	*SFMBT2*
ATF6B	*CR1*	*HLA-DQB1*	*KIR2DL4*	*MIR196A2*	*SFTA2*
ATP6V1G2	*CSF2RB*	*HLA-DRA*	*KIR2DL5A*	*MIR223*	*SKIV2L*
BAT1	*CSNK2B*	*HLA-DRB1*	*KIR2DL5B*	*MRC1*	*SLC11A1*
BAT2	*CTLA4*	*HLA-DRB6*	*KIR2DP1*	*MSH5*	*SLC44A4*
BAT3	*CUBN*	*HLA-DRB9*	*KIR2DS1*	*NCR3*	*SNCA*
BAT4	*CYP21A2*	*HLA-F*	*KIR2DS2*	*NEBL*	*STK19*
BAT5	*CYP2E1*	*HLA-G*	*KIR2DS3*	*NFKBIL1*	*TAP1*
BATF	*DDAH2*	*HSPA1A*	*KIR2DS4*	*NINJ1*	*TAP2*
BATF3	*DDX39B*	*HSPA1B*	*KIR2DS5*	*NLRP1*	*TCF19*
BCHE	*DEC1*	*HSPA1L*	*KIR3DL1*	*NLRP3*	*TGFB1*
BCL10	*DEFB1*	*ICAM1*	*KIR3DL2*	*NOD1*	*TGFBR1*
BRD2	*DOM3Z*	*IFLTD1*	*KIR3DL3*	*NOD2*	*TGFBR2*
BTNL2	*EGFL8*	*IFNG*	*KIR3DP1*	*NOS2*	*TIRAP*
C13orf31	*EHMT2*	*IFNGR1*	*KIR3DS1*	*NOS3*	*TLR1*
C2	*ERBB2*	*IL10*	*LACC1*	*NOTCH4*	*TLR2*
C3	*FAM89A*	*IL10RA*	*LAMA2*	*NOVA2*	*TLR4*
C4B	*FCN1*	*IL10RB*	*LGALS3*	*OCA2*	*TNF*
C6orf10	*FCN2*	*IL12B*	*LRRK2*	*OPA1*	*TNFRSF25*
C6orf15	*FKBPL*	*IL12RB1*	*LSM2*	*PACRG*	*TNFSF15*
C6orf25	*FLOT1*	*IL12RB2*	*LST1*	*PARK2*	*TNFSF8*
C6orf26	*GNG2*	*IL13*	*LTA*	*PBX2*	*TNXB*
C6orf27	*GNL1*	*IL13RA1*	*LTA4H*	*PKD1L1*	*TOLLIP*
C6orf47	*GPR182*	*IL17A*	*LTB*	*PPT2*	*TRIM10*
C6orf48	*GPSM3*	*IL17F*	*LY6G5B*	*PRKCQ*	*TRIM67*
C7orf44	*GSTM1*	*IL18R1*	*LY6G5C*	*PRRT1*	*VARS*
C7orf69	*GTF2H4*	*IL18RAP*	*LY6G6C*	*PSMB8*	*VARS2*
CCDC122	*HCP5*	*IL1R1*	*LY6G6D*	*PSMB9*	*VDR*
CCDC88B	*HLA-A*	*IL2*	*LY6G6F*	*PSORS1C1*	*WASF5P*
CCHCR1	*HLA-B*	*IL23R*	*MAP4K2*	*PSORS1C2*	*ZBTB12*
CCL3	*HLA-C*	*IL4*	*MAPT*	*PTPN22*	*ZNF608*

Note - The reported 227 leprosy susceptible genes were taken from our previous study (Ref. [9] and references therein). The newly identified leprosy risk gene *OPA1* [10] was also included in the analysis.

Table S6. SNP-SNP interaction analysis of *PARL*, *PINK1, LRRK2* and *OPA1* in 527 leprosy patients and 583 controls

CHR1	SNP1	CHR2	SNP2	OR_INT	STAT	*P-value* *
1	rs10916832	3	rs414237	1.488	6.547	0.011
1	rs650616	3	rs7624750	0.640	9.258	0.002
1	rs650616	3	rs9851685	0.700	7.066	0.008
1	rs650616	3	rs4443116	0.756	4.713	0.030
1	rs3738140	12	rs1873613	0.540	4.363	0.037
1	rs3738140	12	rs1427267	0.520	5.295	0.021
1	rs3738140	12	rs7298930	0.542	5.138	0.023
1	rs3738140	12	rs3761863	0.556	4.155	0.042
3	rs2305666	12	rs732374	1.360	5.000	0.025
3	rs9838374	12	rs34778348	4.632	4.848	0.028
3	rs7646539	3	rs100774	0.669	5.692	0.017
3	rs7624750	3	rs100774	0.662	5.013	0.025
3	rs100774	3	rs9851685	0.582	10.080	0.001
3	rs100774	3	rs414237	1.591	6.204	0.013
3	rs100774	3	rs4443116	0.617	8.733	0.003

CHR1, Chromosome of the first SNP; SNP1, Identifier for the first SNP; CHR2, Chromosome of the second SNP; SNP2, Identifier for the second SNP; OR_INT, Odds ratio for interaction; STAT, Chi-square statistic, 1df; *P-value*, asymptotic *P*-value.

* *P*-values of the Bonferroni correction for multiple tests. The significance of the *P*-value should be 1.543×10^{-4}, (324 tests in this analysis). Only *P*-value less than 0.05 were shown here.

The SNP information of *LRRK2* and *OPA1* were taken from our previous studies [10,11].

Table S7. SNaPshot primers for genotyping 5 *PARL* SNPs and 8 *PINK1* SNPs

SNP	Location	Primer (5'-3')
rs1061593 [a]	tagSNPs, 3'UTR, PARL	Forward: ACGGGCTTCCACTTCACA<br>Reverse: TAAATGTGAGTCATTCAATCCCA<br>Probe: ct(gact)$_8$ACAGACCTCCTTATGGCCAAGATGAGCCTC
rs2305666 [a]	tagSNPs, Intron, PARL	Forward: TCTAAAGAGCAGCACATTTTCTAG<br>Reverse: ACCTATTATTGGGGACATAAGTAACT<br>Probe: t(gact)$_7$AGTTTACATGCTGCACATTTCTAGGTGAGC
rs10937153 [a]	tagSNPs, Intron, PARL	Forward: AGGTATTCCTCTACTTGTTGAATTAAAA<br>Reverse: TTATTGAAATCAGTCCTTATTGGC<br>Probe: t(gact)$_6$ATTCCTCCAGTCTCTGTAGGCAACAGGCAA
rs12631031 [a]	tagSNPs, Intron, PARL	Forward: TATTCTTTGATACATGAAGTGGATTT<br>Reverse: TTATCCTCATTTCTCAGATGGG<br>Probe: (gact)$_{11}$ATTCCCGAATCCACCCAGTTCTAGCTGTGT
rs7653061 [a]	tagSNPs, 5'UTR, PARL	Forward: ACCTCTTCCAGGAGGCCT<br>Reverse: TTGCAGAGATAAGCATAAAGCG<br>Probe: act(gact)$_9$CCTTTCCCAGACCTCCACTCCAATTTAGAT
rs10916832 [a]	tagSNPs, 3'UTR, CDA	Forward: TGGGCTGGACCTAACTGC<br>Reverse: AGTAGCTACTGAGAAAACCCTTTGT<br>Probe: act(gact)$_2$TAACCATCCTAGAGTGTGTTTTTGTCTCAT
rs10916840 [a]	tagSNPs, 5'UTR, PINK1	Forward: TGCGTGTGTGTGTTCTGTG<br>Reverse: TTTTGAAGACCCCAAGACAA<br>Probe: (gact)$_1$CTATGCCATTAAACAAACGGTGTGGCTTTG
rs1043424 [a]	tagSNPs, Exon, PINK1	Forward: AAATGTGCTTCATCTAAGCCTC<br>Reverse: AACACTTCTCTGTGAGCCTGTT<br>Probe: GGTGAACATATTCTAGCCCTGAAGA
rs1573132	tagSNPs, 3'UTR, PINK1	Forward: CTATTGCCTAAATCAGCGTCA<br>Reverse: GGTGGGAATCACTGAAATG<br>Probe: ct(gact)$_9$CAACTGAGCTGTTCTAGTTTTCTCT
rs650616	tagSNPs, 3'UTR, PINK1	Forward: TAGTTTTCTCTTCCCCAGCA<br>Reverse: ATACAAGAAAGTTGTTGTTGCTAGTAGA<br>Probe: (gact)$_4$AGCACTGTCATCTAGATTTTCCATTTCAGT
rs607254	tagSNPs, Intron, DDOST	Forward: CAGCAATGAGGAGGGTGTT<br>Reverse: AGGCGTGGCAGCTTTTCC<br>Probe: t(gact)$_8$CATGTGGATACTGGGAACAAAACGA
rs3738140	tagSNPs, Intron, DDOST	Forward: CTCTTGGCTTTTGGCTAAGC<br>Reverse: AAAACGGCTGTCATTGACC<br>Probe: t(gact)$_{13}$TGCCATATGCCCCTGAAACCTGGAA
rs4704 [a]	tagSNPs, Exon, DDOST	Forward: TTGGAGGCAACATCAACG<br>Reverse: ACTCACCAATGTCGGAGCT<br>Probe: t(gact)$_5$CGTGGAGACCATCAGTGCCTTTATTGACGG

Note: (GACT)n, n repeats of "GACT"

[a] These primers for the SnaPShot assay were taken from our recent study [1].

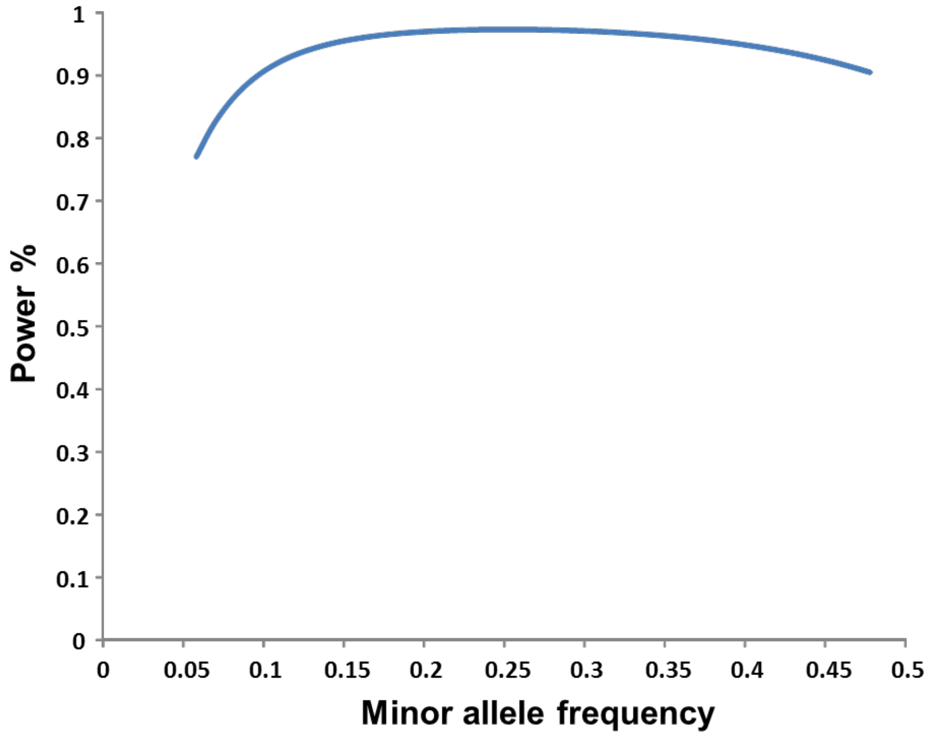

Figure S1. Power estimate for the case-control association analysis (assuming odds ratio value as 1.6; case, n = 527; control, n= 583). Statistical power was computed under the gene only hypothesis and the dominant model by using the Quanto software [12].

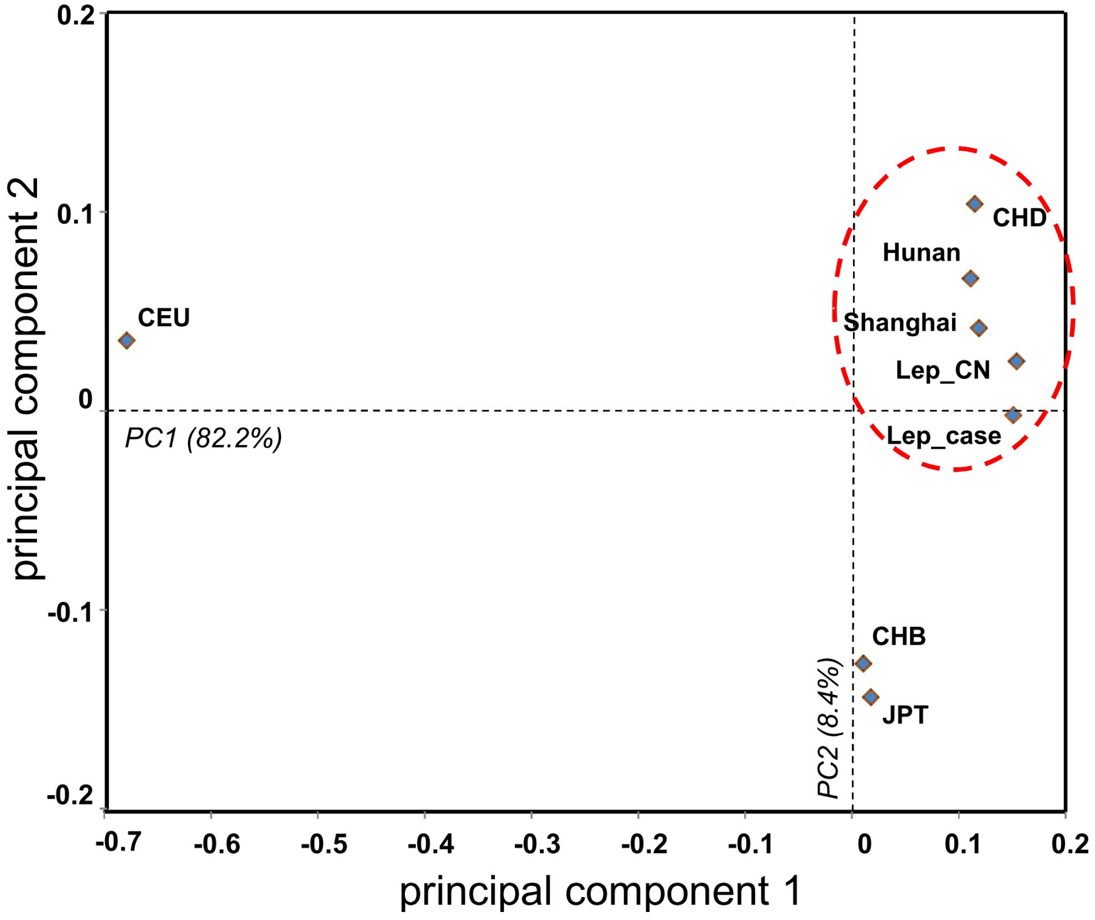

Figure S2. PC map of Han regional populations. It contained the leprosy patient (Lep_case), Yuxi control (Lep_CN) population, reported Han Chinese populations (Hunan and Shanghai)[1] and other populations from HapMap database (CHB, CHD, JPT and CEU) [13]. CHB: Han Chinese in Beijing, China (n=136); CHD: Chinese in Metropolitan Denver, Colorado (n=109); JPT: Japanese in Tokyo, Japan (n=113); CEU: Utah residents with Northern and Western European ancestry (n=113).

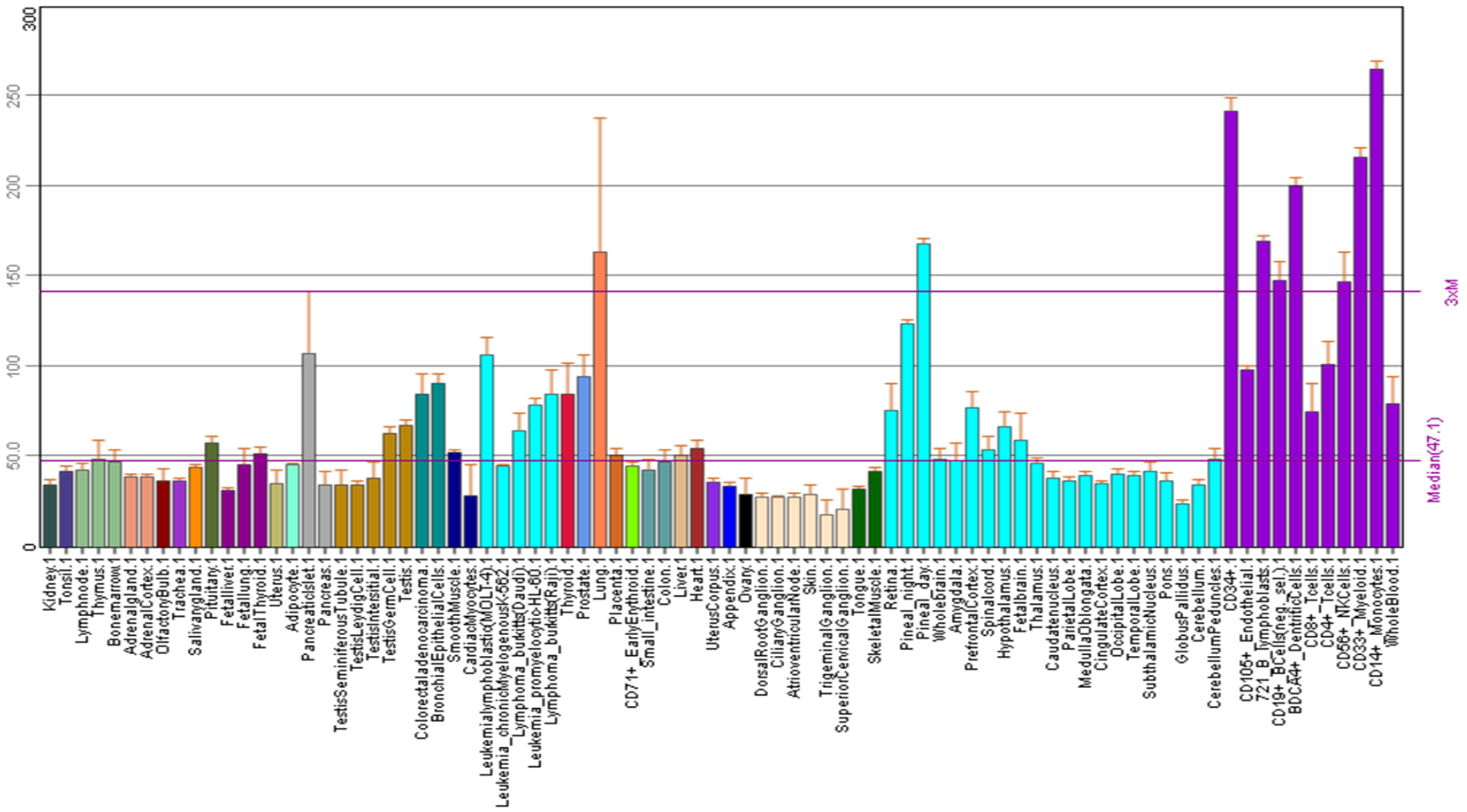

Figure S3. mRNA expression pattern of *PARL* in human tissues. *PARL* is widely expressed in human tissues, especially in immune cells. Data were retrieved from the BioGPS (http://biogps.org/#goto=welcome) [14].

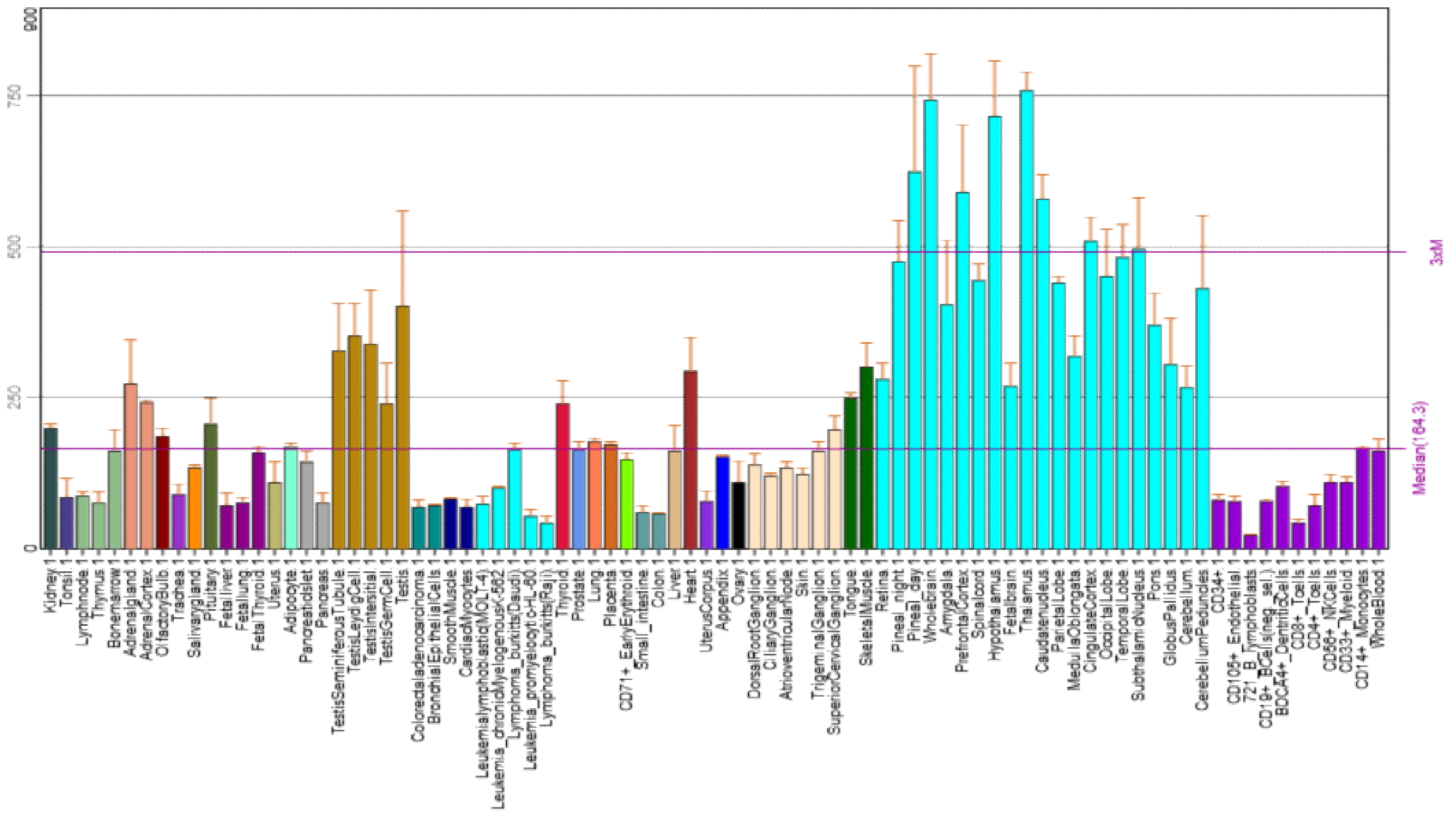

Figure S4. mRNA expression pattern of *PINK1* in human tissues. *PINK1* is widely expressed in human tissues, especially in central nervous system. Data were retrieved from the BioGPS (http://biogps.org/#goto=welcome) [14].

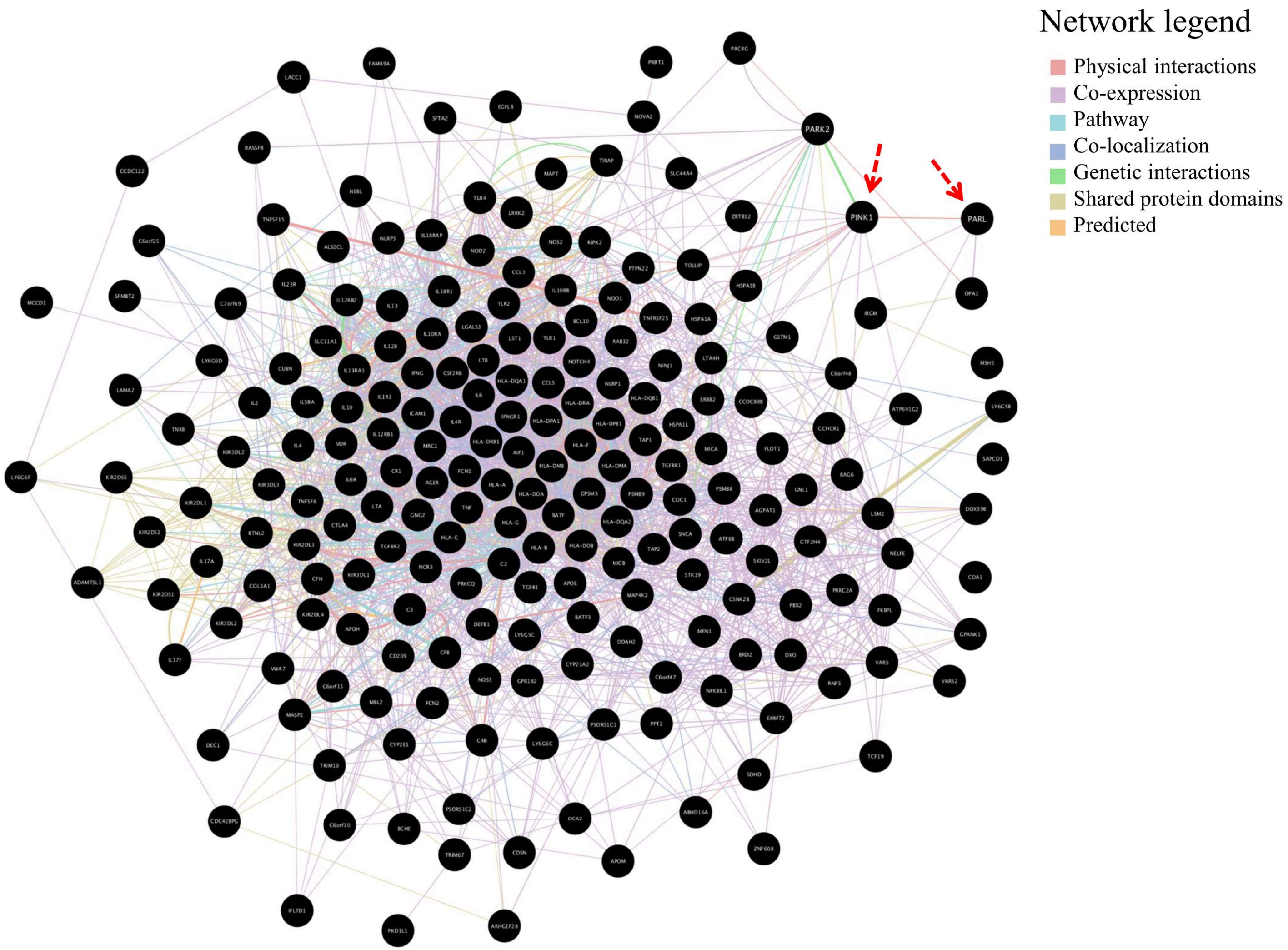

Figure S5. Protein interaction network of PARL, PINK1 and reported leprosy susceptibility genes (Ref. [9] and references therein; Table S5) by using the GeneMANIA prediction server (http://www.genemania.org/) [15]. The PARL and PINK1 proteins were marked by red arrows.

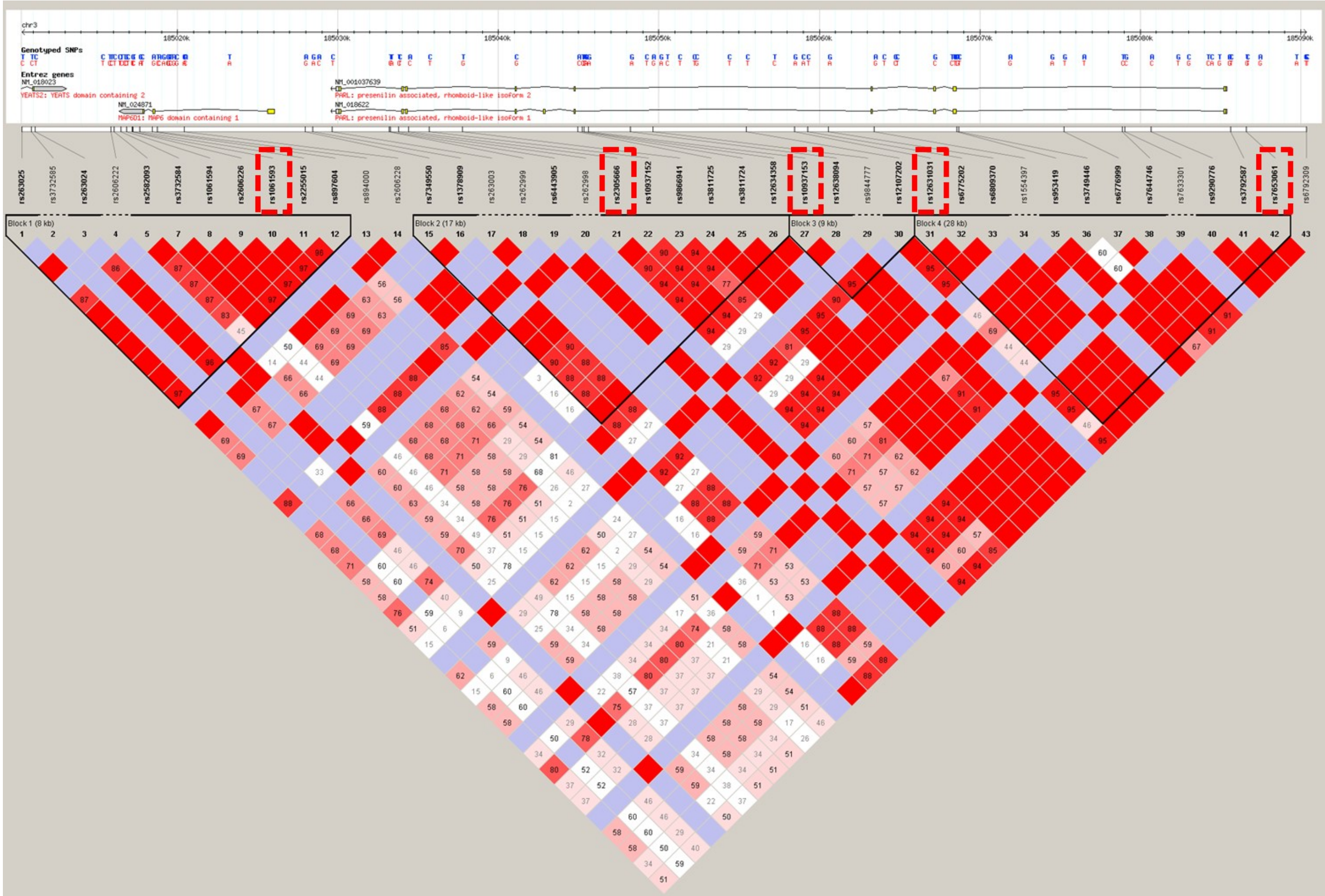

Figure S6. Linkage disequilibrium plot of the *PARL* SNPs based on CHB, HapMap, Phase 3 [13]. SNPs selected for genotyping in this study were marked in red box.

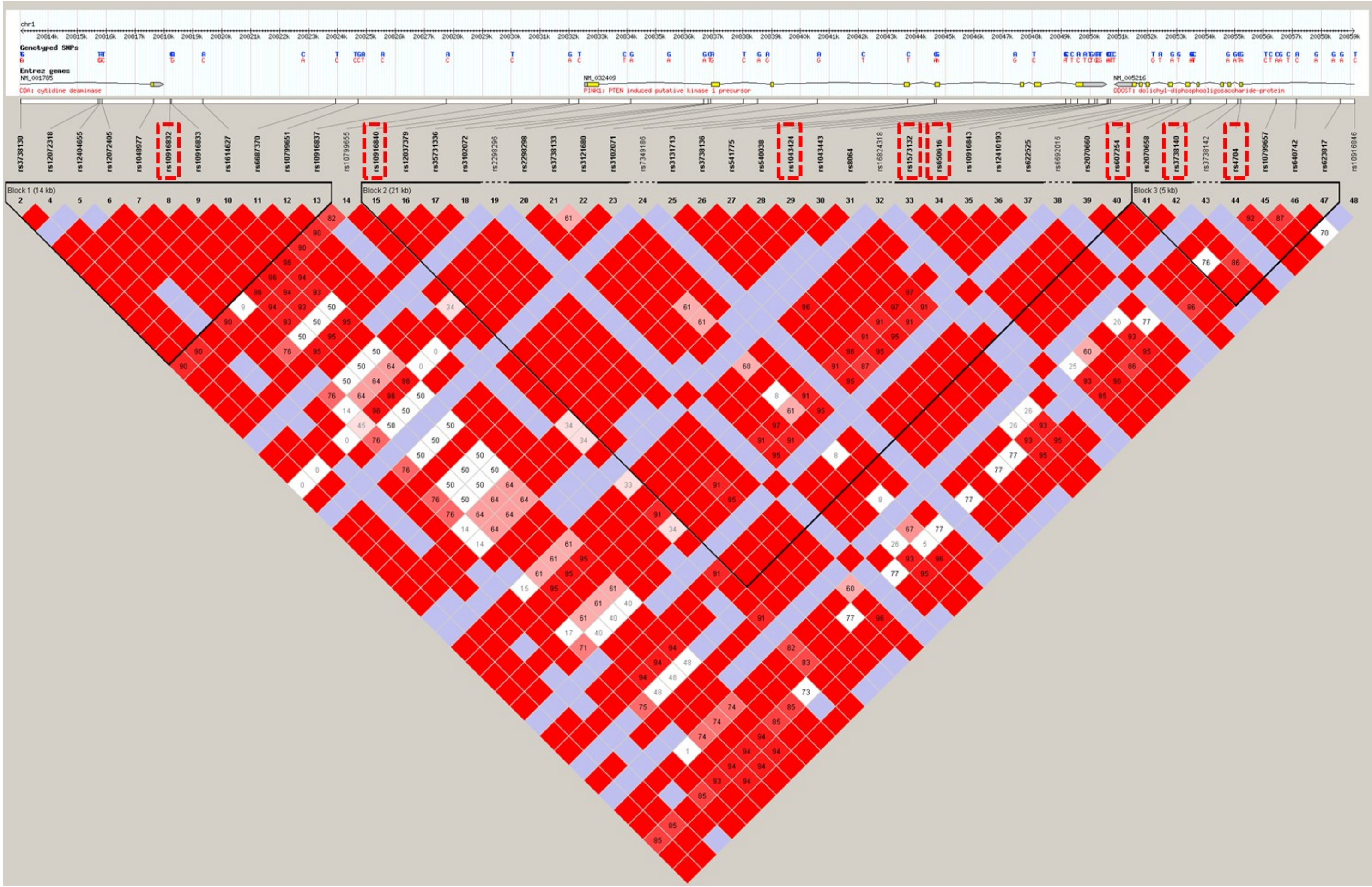

Figure S7. Linkage disequilibrium plot of the *PINK1* SNPs based on CHB, HapMap, Phase 3 [13]. SNPs selected for genotyping in this study were marked in red box.

Supplementary References

1 Li, X. *et al.* Common variants of the PINK1 and PARL genes do not confer genetic susceptibility to schizophrenia in Han Chinese. *Molecular genetics and genomics : MGG* **290**, 585-592, doi:10.1007/s00438-014-0942-1 (2015).

2 1000 Genomes Project Consortium *et al.* A global reference for human genetic variation. *Nature* **526**, 68-74, doi:10.1038/nature15393 (2015).

3 Kumar, P., Henikoff, S. & Ng, P. C. Predicting the effects of coding non-synonymous variants on protein function using the SIFT algorithm. *Nature protocols* **4**, 1073-1081, doi:10.1038/nprot.2009.86 (2009).

4 Ng, P. C. & Henikoff, S. SIFT: Predicting amino acid changes that affect protein function. *Nucleic acids research* **31**, 3812-3814 (2003).

5 Adzhubei, I. A. *et al.* A method and server for predicting damaging missense mutations. *Nature methods* **7**, 248-249, doi:10.1038/nmeth0410-248 (2010).

6 Chun, S. & Fay, J. C. Identification of deleterious mutations within three human genomes. *Genome research* **19**, 1553-1561, doi:10.1101/gr.092619.109 (2009).

7 Schwarz, J. M., Cooper, D. N., Schuelke, M. & Seelow, D. MutationTaster2: mutation prediction for the deep-sequencing age. *Nature methods* **11**, 361-362, doi:10.1038/nmeth.2890 (2014).

8 Belone Ade, F. *et al.* Genome-Wide Screening of mRNA Expression in Leprosy Patients. *Frontiers in genetics* **6**, 334, doi:10.3389/fgene.2015.00334 (2015).

9 Zhang, D.-F., Wang, D., Li, Y.-Y. & Yao, Y.-G. Integrative analyses of leprosy susceptibility genes indicate a common autoimmune profile. *Journal of dermatological science* **82**, 18-27, doi:10.1016/j.jdermsci.2016.01.001 (2016).

10 Xiang, Y.-L., Zhang, D.-F., Wang, D., Li, Y.-Y. & Yao, Y.-G. Common variants of OPA1 conferring genetic susceptibility to leprosy in Han Chinese from Southwest China. *Journal of dermatological science* **80**, 133-141, doi:10.1016/j.jdermsci.2015.09.001 (2015).

11 Wang, D. *et al.* Association of the LRRK2 genetic polymorphisms with leprosy in Han Chinese from Southwest China. *Genes and immunity* **16**, 112-119, doi:10.1038/gene.2014.72 (2015).

12 Gauderman, W. J. Sample size requirements for matched case-control studies of gene-environment interaction. *Stat Med* **21**, 35-50, doi:10.1002/sim.973 (2002).

13 International HapMap, C. The International HapMap Project. *Nature* **426**, 789-796, doi:10.1038/nature02168 (2003).

14 Wu, C., Jin, X., Tsueng, G., Afrasiabi, C. & Su, A. I. BioGPS: building your own mash-up of gene annotations and expression profiles. *Nucleic acids research* **44**, D313-316, doi:10.1093/nar/gkv1104 (2016).

15 Warde-Farley, D. *et al.* The GeneMANIA prediction server: biological network integration for gene prioritization and predicting gene function. *Nucleic acids research* **38**, W214-220, doi:10.1093/nar/gkq537 (2010).
